# Nursing interventions promoting parenting during child hospitalization: a scoping review

**DOI:** 10.1590/0034-7167-2025-0103

**Published:** 2026-02-23

**Authors:** Joana Rita Guarda da Venda Rodrigues, Bárbara Rodrigues e Sousa, Francisca Cardoso Macedo, Mariana Fidalgo Cabouco, Mariana Marreiros da Rocha, Tatiana Filipa Tiago Carneiro

**Affiliations:** I Escola Superior de Enfermagem de Lisboa. Lisboa, Portugal; II Centro de Investigação, Inovação e Desenvolvimento em Enfermagem de Lisboa. Lisboa, Portugal.; III Unidade Local de Saúde Lisboa Ocidental, Hospital de S. Francisco Xavier. Lisboa, Portugal.; IV Unidade Local de Saúde Arco Ribeirinho, Hospital de Nossa Senhora do Rosário. Barreiro, Portugal.

**Keywords:** Parenting, Pediatric Nursing, Family, Child, Hospitalization

## Abstract

**Objectives:**

to map knowledge about nursing interventions that promote parenting during child hospitalization.

**Methods:**

a scoping review conducted according to the JBI methodology and the Preferred Reporting Items for Systematic Reviews and Meta-Analyses extension for Scoping Reviews checklist.

**Results:**

a total of 1,491 publications were identified, including 17 studies. It was evident that primary nursing interventions, which promote child hospitalization, focus on health promotion and parental education. Secondary nursing interventions focus on parental empowerment, emotional support, and maintaining family bonds. Tertiary interventions focus on connecting with community resources.

**Final Considerations:**

the mapped nursing interventions, based on a partnership model in care and a family-centered approach, demonstrate potential for responding to parents’ needs and promoting parenting during the hospitalization process of children in conditions of increased vulnerability.

## INTRODUCTION

Parenting is an essential component of a child’s healthy development, enabling secure attachment^(1)^. Parenting is defined by the International Council of Nurses as caring, in which parents assume the responsibilities of being a mother/father, adopting behaviors designed to facilitate the incorporation of a newborn into the family unit, and optimizing child growth and development, internalizing individuals’, families’, friends’, and society’s expectations regarding appropriate or inappropriate parental role behaviors^(2)^.

Hospitalization is widely portrayed in the literature as a stressful time for children and their families^(3,4)^ whether due to confirmation of a diagnosis or worsening of a clinical situation, thus resulting in the possibility of adverse events occurring, in a situation of increased vulnerability^(4)^, both for children and their family.

In this context, vulnerability, understood as a dynamic and procedural phenomenon^(5)^associated with fragility or the risk of suffering harm, often present in situations of exposure to multicausal and intersectoral adverse conditions or experiences, which represent a threat to child development^(6)^, can be mitigated by family–centered interventions^(6)^, with a focus on promoting parenting skills^(5)^.

For Betty Neuman, nursing interventions are defined as intentional actions that help the client maintain or achieve system stability^(7)^. The author identifies three levels of interventions: primary, secondary, and tertiary^(7)^. Primary interventions are developed when a stressor is suspected, i.e., no reaction has yet occurred, but the risk of it occurring is known^(7)^. On the other hand, secondary interventions “(...) involve interventions or treatments initiated after the occurrence of stress symptoms”, in an attempt to stabilize the system^(7)^. Finally, tertiary interventions occur after active treatment and focus on system readjustment, with the goal of regaining stability^(7)^.

Nursing interventions during child hospitalization and their families can enhance parental self–confidence, promoting emotional regulation and reorganization in the face of the new scenario of their child hospitalization^(8)^. By creating opportunities that highlight parental skills, these interventions contribute to the acquisition of new abilities, which allow parents to respond more appropriately to their children’s needs^(7)^. In this hospitalization context, family–centered care and partnership care emerge as central pillars of intervention^(9)^. The philosophy of family–centered care advocates for the comprehensive role of the family in children’s lives, recognizing it as an essential and comprehensive element of care and identifying empowerment and empowerment as fundamental concepts^(10)^. The Institute for Patient–and Family–Centered Care adds that respect, dignity, information sharing, participation, and collaboration are essential concepts for the development of a practice sustained in family–centered care^(11)^. In turn, the partnership model in care developed by Anne Casey in 1988 is based on the recognition and respect for the role of the family in child care^(12)^.

There are several studies on child hospitalization, especially focusing on premature newborns hospitalized in a Neonatal Intensive Care Unit^(1)^. A scoping review developed by Barros *et al* ^(13)^. focuses only on nursing interventions that promote the adaptation of children/young people/families to hospitalization. In turn, a review developed by Querido *et al*.^(1)^ only focuses on nursing interventions that promote attachment to hospitalized newborns, and it was found that the vulnerable situations described are mostly related to child hospitalization. Prematurity and contact with opioids in the perinatal period are also identified as situations that increase vulnerability.

This review, in turn, focuses both on aspects related to parenting in hospital settings and on strengthening the bond and parenting skills, regardless of children’s age or the reason for hospitalization, which was not explored in the previously mentioned reviews. Also, based on the preliminary literature search conducted in the JBI, Prospero, and Open Science Framework (OSF) databases in March 2024, no reviews focusing on nursing interventions that promote parenting during vulnerable child hospitalization were identified. Concomitantly, the literature on the phenomenon under study was dispersed. Thus, identifying available scientific evidence will allow us to synthesize knowledge about nursing interventions that promote parenting during child hospitalization, guiding practice and highlighting gaps for future research.

In convergence with the above, this review aimed to map knowledge about nursing interventions that promote parenting during child hospitalization, answering the question “What are the nursing interventions that promote the exercise of parenting during a child hospitalization?” and the sub-questions “What are the vulnerable situations experienced by parents and family during hospitalization?” and “What are the parents’ and family’s needs during a child hospitalization?”.

## OBJECTIVES

To map knowledge about nursing interventions that promote parenting during child hospitalization.

## METHODS

This scoping review was carried out according to the methodology recommended by JBI and in accordance with Preferred Reporting Items for Systematic reviews and Meta-Analyses extension for Scoping Reviews (PRISMA-ScR)^(14)^.

### Eligibility criteria

Eligibility criteria for the selected studies were defined based on the mnemonic PCC (Population, Concept, and Context). Thus, the Population (P) of this scoping review includes parents and families of hospitalized children who are in vulnerable situations; the Concept (C) corresponds to nursing interventions that promote parenting in vulnerable situations; and the Context (C) is child hospitalization.

This review included primary studies addressing nursing interventions that promoted parenting during child hospitalization. Studies focused solely on nursing interventions focused on bonding with hospitalized newborns were excluded. No time limit was established. The protocol was written, verified, and reviewed by the authors and was prospectively registered with the OSF (https://osf.io/vjdb5/).

### Research strategy and study identification

To identify the studies, the MEDLINE (via PubMed), CINAHL Unlimited (via EBSCO), Scopus, Psychology Behavioral Science Collection, MedicLatina, Web of Science, Cochrane Central Register of Controlled Trials, Cochrane Database of Systematic Reviews, Cochrane Clinical Answers, and ERIC electronic databases were used. To identify unpublished studies, a search was conducted in the *Repositórios Científicos de Acesso Aberto de Portugal* (RCAAP), Google Scholar, and OpenAIRE.

The research began on March 20, 2024, with a search of the MEDLINE (via PubMed) and CINAHL Unlimited (via EBSCO) electronic databases. Natural language search terms were used to help identify the keywords used in titles and abstracts, as well as the indexing terms. Subsequently, natural language words, keywords, and indexing terms listed were combined with Boolean operators and, where possible, an asterisk (B) operator to form the search expression (TI ParentB OR TI Family OR AB ParentB OR AB Family) AND (TI PediatricB OR TI NursB OR TI NeonatalB OR TI HospitalB OR AB PediatricB OR AB NursB OR AB NeonatalB OR AB HospitalB) AND (TI Vulnerability OR TI “Special Populations” OR AB Vulnerability OR AB “Special Populations”)), which was adapted to the specificities of each database or repository.

Then, the bibliographic references in the previously identified records were analyzed. Unpublished studies and gray literature were also searched in RCAAP, Google Scholar, and OpenAIRE.

### Data extraction, analysis and synthesis

The search results were exported to the Mendeley Desktop reference manager (version 1.19.8), through which duplicate records were identified and removed. To aid article selection, the records were then entered into the Qatar Computing Research Institute (Rayyan QCRI) platform. Two reviewers independently and blindly assessed the eligibility of documents by assessing the titles and abstracts against inclusion and exclusion criteria. A third reviewer was consulted to clarify any discrepancies. Articles that met the eligibility criteria were retrieved in full, and the full text was assessed in detail for eligibility by two or more reviewers independently and blindly. Whenever the full text was not available, the corresponding author was contacted and/or the article was requested from a university library.

Descriptive analysis and synthesis of data from records that met the inclusion and exclusion criteria were performed independently and blindly by two or more authors, using self-developed frameworks. These frameworks included dimensions characterizing the selected publications and studies, as well as vulnerability, parents’ needs, and nursing interventions, in line with the objectives and the scoping review question. After mutual review of records, the authors identified and highlighted areas of uncertainty. Any doubts were resolved through discussion, with a third reviewer being consulted to clarify disagreements, culminating in the presentation of agreed-upon results.

## RESULTS

**Figure 1:**
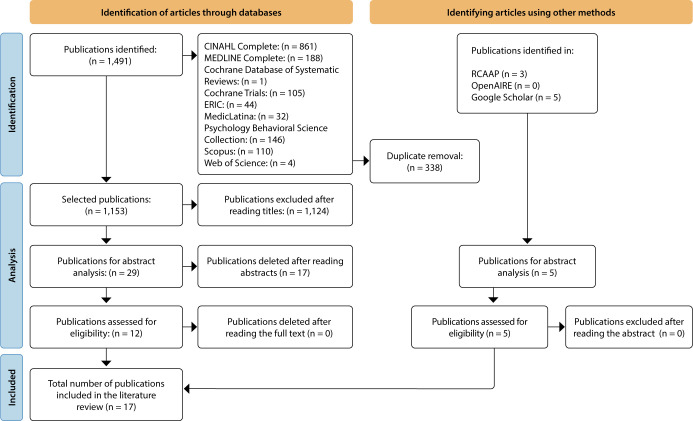
Preferred Reporting Items for Systematic reviews and Meta-Analyses extension for Scoping Reviews (adapted) flowchart of the study selection process, Lisbon, Portugal

The search carried out in the databases allowed the identification of 1,491 publications, of which 17 articles were included. The results obtained in the different stages of study selection are presented according to the PRISMA-ScR recommendations, explained in Figure 1.

**Figure 2:**
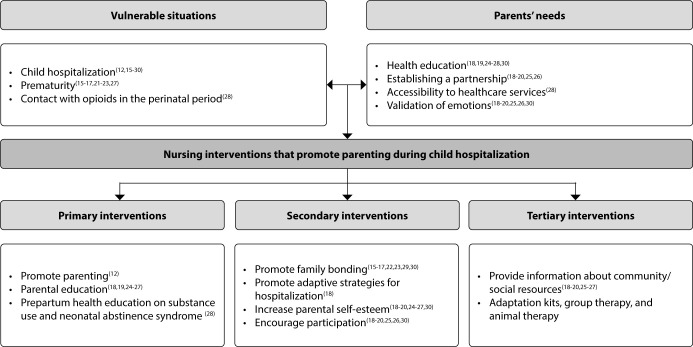
Summary diagram of the results obtained, Lisbon, Portugal

The selection process resulted in 17 studies, which were included in this scoping review. The results are summarized and organized in Charts 1 and 2, and in Figure 2, respectively.

**Chart 1:** General characterization of studies included in the scoping review, Lisbon, Portugal

Study number (S) Title | author(s)	Country | year	Study design	Objective(s)	Participants | characterization
S1 Parent Visiting and Participation in Infant Caregiving Activities in a Neonatal Unit Franck *et al*.^(15)^	United Kingdom, 2003	Qualitative	Describe the frequency and duration of parental visits and participation in care, and identify factors associated with parental involvement.	110 parents visiting their babies in a neonatal unit.
S2 Adjusting to being a father to an infant born prematurely: experiences from Swedish fathers Lindberg *et al*.^(16)^	Sweden, 2008	Qualitative	Describe the experiences of being a parent of a premature newborn.	Eight parents of premature babies cared for in a Neonatal Intensive Care Unit in northern Sweden.
S3 The parent–nurse relationship in the neonatal intensive care unit context – closeness and emotional involvement Fegran *et al*.^(17)^	Norway, 2009	Qualitative	Explore the experiences of parents and nurses regarding their relationship when their premature child is hospitalized.	Six mothers, six fathers, and six nurses in a Neonatal Intensive Care Unit in Norway.
S4 Percepções da família acerca das dificuldades de adaptação da criança à hospitalização: subsídios para a enfermagem Gomes *et al*.^(18)^	Brazil, 2013	Qualitative exploratory- descriptive	Explore family perceptions regarding the child’s difficulties in adapting during hospitalization.	15 family members of three hospitalized children.
S5 Validation of Hospitalization Impact Scale among families with children hospitalized for cancer treatment Lyu *et al*.^(19)^	China, 2015	Quantitative and qualitative	Modify and validate the psychometric properties of the Hospitalization Impact Scale to assess the impact of hospitalization of children with cancer on their families.	253 families with children hospitalized for cancer treatment in four pediatric oncology departments in hospitals in China.
S6 Interactions Between Children And Pediatric Nurses At The Emergency Department: A Swedish Interview Study Grahn *et al*.^(20)^	Sweden, 2016	Qualitative	Describe strategies used by nurses when interacting with children aged 3 to 6.	Seven nurses with experience in pediatric care.
S7 Differences And Similarities Between Mothers And Fathers Of Premature Children: A Qualitative Study Of Parents’ Coping Experiences In A Neonatal Intensive Care Unit Hagen *et al*.^(21)^	Norway, 2016	Qualitative	Explore and describe the coping experiences of parents of children admitted to a Neonatal Intensive Care Unit.	Eight mothers and eight fathers who were interviewed between one and six months after discharge from the Neonatal Intensive Care Unit.
S8 A Life Uncertain - My Baby’s Vulnerability: Mothers’ Lived Experience Of Connection With Their Preterm Infants In A Botswana Neonatal Intensive Care Unit Ncube *et al*.^(22)^	Botswana, 2016	Qualitative	Explore and describe the experiences of mothers regarding the care of premature infants hospitalized in a Neonatal Intensive Care Unit.	Eight mothers of premature babies who were admitted to a neonatal unit immediately after birth.
S9 Supporting Of The Fathers To Visit Their Infants In Neonatal Intensive Care Unit Decreases Their Stress Level: A Pretest–Posttest Quasi- Experimental Study Özdemir & Alemdar^(23)^	Turkey, 2016	Quasi- experimental	Determine the effect of parental support and visits to their babies in the Neonatal Intensive Care Unit on parental stress levels.	47 fathers of babies admitted to a Neonatal Intensive Care Unit in a hospital in eastern Turkey.
S10 The Lived Experience of Jordanian Parents In a Neonatal Intensive Care Unit: A Phenomenological Study Abuidhail *et al*.^(24)^	Jordan, 2017	Qualitative	Describe the lived experiences, care needs and support systems of parents of newborns admitted to a Neonatal Intensive Care Unit.	Ten participants: eight mothers and two fathers of newborns in the Neonatal Intensive Care Unit.
S11 Experiences and needs of parents of critically injured children during the acute hospital phase: A qualitative investigation Foster *et al*.^(25)^	Australia, 2017	Qualitative, part of mixed longitudinal study	Explore the experiences of parents of critically injured children during the acute phase of hospitalization and determine their support needs during this period.	40 parents of 30 critically injured children, ages 1 to 13.
S12 Family Stress in Pediatric Critical Care Hagstrom^(26)^	United States of America, 2017	Qualitative and quantitative	Describe the sources of stress for families whose children have been hospitalized in the Pediatric Intensive Care Unit for more than a week.	Nine parents (eight mothers and one father) from eight families, all married.
S13 Sistemas de apoio na unidade de terapia intensiva pediátrica: perspectiva dos familiares Bazzan *et al*.^(27)^	Brazil, 2019	Qualitative	Identify and analyze the support systems used by family members as they adjust to their child hospitalization in the Pediatric Intensive Care Unit.	13 family members of children admitted to the Pediatric Intensive Care Unit.
S14 Engaging Mothers to Implement Nonpharmacological Care for Infants with Neonatal Abstinence Syndrome: Perceptions of Perinatal and Pediatric Nurses Shuman *et al*.^(28)^	United States of America, 2020	Qualitative	Describe pediatric and perinatal nurses’ perceptions of maternal participation in the care of opioid-exposed infants, as well as the facilitators and barriers to participation.	21 nurses from the delivery room, the Neonatal Intensive Care Unit and the pediatrics service.
S15 Parental presence, participation, and engagement in paediatric hospital care: A conceptual delineation Harlow *et al*.^(29)^	United States of America, 2023	Conceptual analysis	Outline the concepts of parental presence, participation, and engagement in pediatric hospital care.	Publications between 1991 and 2003.
S16 O exercício parental durante a hospitalização do filho: modelo de intencionalidades terapêuticas de enfermagem face à parceria de cuidados Sousa *et al*.^(12)^	Portugal, 2023	Action research	Identify nurses’ therapeutic intentions when promoting partnership care with parents during the child hospitalization.	Nurses from the pediatric inpatient department at *Hospital Pedro Hispano*, parents, and children hospitalized there.
S17 Mother’s mental health and the interaction with her moderate preterm baby in the NICU Mira *et al*.^(30)^	Chile, 2024	Correlational	Describe the impact of having a moderately preterm newborn hospitalized in a Neonatal Intensive Care Unit on the mothers’ mental health and its relationship with dyad interaction.	85 moderately preterm mother- newborn dyads.

**Chart 2:** Description of vulnerable situations, parents’ needs, and nursing interventions that promote parenting performance during child hospitalization, Lisbon, Portugal

Study number Author(s) Year	Vulnerable situation	Parental and/or family needs	Nursing Interventions
S1 Franck et al. (2003)^(15)^	Child hospitalization Prematurity	Parents, particularly mothers, need emotional and practical support during their baby’s hospitalization, as well as to actively participate in newborn care, in order to foster emotional bonding and reduce stress.	Encourage parental participation in child care; Promote physical closeness; Promote breastfeeding; Provide emotional support to parents; Communicate with parents; Participate in routine care.
S2 Lindberg *et al*. (2008)^(16)^	Child hospitalization Prematurity	Parents of premature newborns expressed a need for emotional and practical support, including education about infant care and validation of emotions such as guilt and anxiety.	Encourage parental involvement in child care; Promote breastfeeding; Establish a partnership; Provide emotional support; Accompany newborns on their first visit; Promote closeness, contact, touch, warmth, and scent through voice; Promote skin-to-skin contact through the kangaroo method; Plan caregiving with parents.
S3 Fegran *et al*. (2009)^(17)^	Child hospitalization	Parents express a need for emotional support and respect as partners in child care, also expressing a desire to be understood and have their emotions validated.	Encourage parental involvement in child care; Promote physical closeness between parents and newborns through touch, skin-to-skin contact, and massage; Strengthen parenting skills and social and psychological development; Establish good communication, providing all information regarding the newborn throughout the different stages of hospitalization; Provide emotional support; Promote the kangaroo method; Encourage mothers to express milk with their newborns; Involve parents in newborn care.
S4 Gomes *et al*. (2013)^(18)^	Child hospitalization	Families require emotional and practical support to help their child adjust to hospitalization.	Communicate effectively with the family; Educate parents on parenting skills and child care knowledge; Promote relaxation or distraction through activities; Encourage adaptive emotional expression; Help parents identify social support resources.
S5 Lyu *et al*.(2015)^(19)^	Child hospitalization	Parents need emotional and practical support, including identifying specific stressors during hospitalization, and interventions targeted to their needs, such as identifying specific stressors during hospitalization and finding interventions that meet their needs.	Communicate effectively with the family; Educate parents on parenting skills and child care knowledge; Help parents identify social support resources.
S6 Grahn *et al*. (2016)^(20)^	Child hospitalization	There is a need for interactions between nurses and children in the emergency department that promote a safe and welcoming environment, valuing communication and parental participation to reduce anxiety and improve the care experience.	Communicate effectively with the family; Educate about parenting skills; Share knowledge about child care; Promote relaxation or distraction through activities; Teach parents strategies for managing anxiety and stress; Encourage adaptive emotional expression; Help parents identify social support resources.
S7 Hagen *et al*. (2016)^(21)^	Child hospitalization Prematurity	Parents expressed the need for emotional support and clear information about their children’s health. They reported the importance of being heard regarding the care of their premature newborn and having their partner present to improve coping.	Encourage parental participation in child care; Promote physical closeness between parents and newborns through touch, skin-to-skin contact, and massage; Communicate with parents; Provide emotional support to parents; Promote parental participation in routine care.
S8 Ncube *et al*. (2016)^(22)^	Child hospitalization Prematurity	Mothers of premature newborns require emotional support, clear information about their child’s health, and opportunities to actively participate in care.	Encourage parental involvement in child care; Promote physical closeness between parents and newborns through touch, skin-to-skin contact, and massage; Promote breastfeeding; Strengthen parenting skills and social and psychological development; Establish a partnership; Establish good communication, providing all information regarding the newborn throughout the different stages of hospitalization; Accompany newborns on their first visit; Promote the kangaroo method; Encourage mothers to express milk with their newborns; Plan care with parents.
S9 Özdemir & Alemdar (2016)^(23)^	Child hospitalization Prematurity	Parents need emotional support and clear information about their child’s condition as a strategy to reduce stress and promote adaptation.	Encourage parental involvement in child care; Promote physical closeness; Communicate with parents; Provide emotional support to parents; Promote parental involvement in routine care.
S10 Abuidhail *et al*. (2017)^(15)^	Child hospitalization	Parents report a need for information and intervention from nurses to cope with stressful situations, as well as emotional and psychological support.	Communicate effectively with the family; Educate on parenting skills and child care knowledge; Empower parents to manage anxiety and stress; Encourage adaptive emotional expression.
S11 Foster *et al*. (2017)^(24)^	Child hospitalization	Parents require psychological support and clear information about their children’s treatment and recovery, and they also express concerns about the emotional impact of injuries on their children and on family dynamics.	Communicate effectively with the family; Educate parents on parenting skills and child care knowledge; Help parents identify social support resources.
S12 Hagstrom (2017)^(26)^	Child hospitalization	Parents express a need for emotional and practical support, including validation of emotions such as guilt and remorse, and the need to maintain connection with their children at home. They also feel a need for clear and honest information about their children’s health and what to expect during hospitalization.	Communicate effectively between healthcare professionals and families; Educate parents on parenting skills and child care knowledge; Promote relaxation or distraction through activities; Empower parents to manage anxiety and stress; Encourage adaptive emotional expression; Help parents identify social support resources.
S13 Bazzan *et al*. (2019)^(27)^	Child hospitalization	Families need a robust support system that includes emotional support from family, friends, and healthcare professionals, as well as interactions with other family members of hospitalized children.	Communicate effectively with the family; Educate parents on parenting skills and child care knowledge; Help parents identify social support resources.
S14 Shuman *et al*. (2020)^(28)^	Contact with opioids in the perinatal period	Help with newborn care is needed if they live far away, lack transportation, have visiting time limits, or have other children.	Educate before birth; Validate mothers’ emotions (remorse and guilt); Help mothers focus on what they can do in the present moment; Communicate objectively and honestly about their substance use history and the baby’s withdrawal symptoms.
S15 Harlow *et al*. (2023)^(29)^	Child hospitalization	Be physically present to accompany their children during hospitalization.	Encourage the physical presence of parents and, if this is not possible, resort to virtual presence through video or phone calls; Encourage parental participation in child care, particularly with feeding, diaper changes, and comfort; Empower parents to participate in care by establishing a relationship that promotes parental education and participation; Encourage the kangaroo care technique and presence with children.
S16 Sousa *et al*. (2023)^(12)^	Child hospitalization	Involvement, or not, in the care process, according to the parents’ needs. Establishing therapeutic relationships with nurses.	Promote parental involvement in development-promoting care; Promote parenting skills to provide complex care; Reduce the level of burnout among parents of children with permanent special needs; Prepare parents to promote child autonomy.
S17 Mira *et al*. (2024)^(30)^	Child hospitalization Prematurity	Need for education and support during hospitalization, as well as unlimited access to the Neonatal Intensive Care Unit.	Facilitate early and frequent skin-to-skin contact for at least 60 minutes to reduce parental and child stress; Promote breastfeeding.

The 17 studies included in this scoping review were conducted between 2003 and 2024 in 12 different countries: the United States of America (3); Brazil (2); Norway (2); Sweden (2); Chile (1); Portugal (1); Australia (1); China (1); Jordan (1); Botswana (1); Turkey (1); and the United Kingdom (1). Concerning study design, 11 qualitative studies, two mixed-method studies, one correlational study, one action research study, one quasi–experimental study, and one conceptual analysis were identified.

From the review of the selected and analyzed articles, pertinent information emerged regarding: i) situations of child vulnerability; ii) parents’ needs; iii) nursing interventions that promote parenting during child hospitalization in vulnerable situations, which respond to the objectives initially established, as can be seen in Chart 2 and Figure 2.

## DISCUSSION

Parenting can be understood as assuming the responsibilities of being a parent during the child’s development(^12^). A child’s hospitalization represents a critical event and a situation of vulnerability in the exercise of parenting(^12^). During their child’s hospitalization, parents experience not only environmental stress, but also complications or uncertainties regarding children’s health status, physical and emotional separation from their baby, and increased levels of parental stress(^30^). According to the study developed by Mira, Coo, and Bastías, which aimed to describe the impact of premature newborn hospitalization on maternal mental health, approximately 92% of mothers reported that hospitalization in the Neonatal Intensive Care Unit was a stressful experience, with parenting being the main source of stress(^30^).

Parents’ attitudes toward their children’s hospitalization depend on the personal characteristics of children, the parents themselves, and nurses, as establishing a partnership between nurses and parents is a path to achieving better outcomes(^12^). Thus, nurses’ ability to intervene in partnership with parents is an essential competency for achieving higher standards of quality in nursing care(^12^).

### Vulnerable situations experienced by children and families

The vulnerable situations in hospitals identified in the included studies are, for the most part, related to child hospitalization itself(^18 # 21, 24 # 27^). Hospitalization appears to alter children’s adaptive capacity, increasing their vulnerability(^18 # 21, 24 # 27^). Prematurity has also been identified as a situation of vulnerability, since preterm newborns have a greater risk of developing neurobehavioral problems compared to full-term newborns(^30^). In addition to the situations mentioned above, contact with opioids in the perinatal period has also been identified as a situation of vulnerability experienced by children(^28^).

### Parents’ needs during child hospitalization in vulnerable situations

The analysis of selected studies highlighted various needs of parents during their children’s hospitalization. Some authors focus on the need for health education, particularly regarding breastfeeding, child development and care, as well as the management of anxiety, stress, and symptoms associated with withdrawal syndrome^(30)^. The importance of establishing a partnership with healthcare professionals, particularly nurses, in providing care to children during hospitalization is also highlighted^(18–20,25,26^).

Likewise, the studies identified show that many parents have difficulty accessing services, either because they live far away or because they do not have access to transportation, which can compromise the establishment of a therapeutic relationship with health teams and participation in child care^(28)^. Parents also express the need to have their emotions validated by nurses in order to consolidate the therapeutic relationship on an empathic basis^(18,19,25,26)^.

### Nursing interventions that promote parenting during child hospitalization in vulnerable situations

#### Primary interventions

Primary nursing interventions focus on promoting parenting, reinforcing parenting practices during hospitalization, ensuring their continuity, and preparing parents to foster their child’s autonomy^(12)^. Among the primary interventions identified, parental education^(18),19,25–27)^) and health education in the prepartum period stand out, particularly regarding maternal substance use and newborn withdrawal symptoms(^28)^.

#### Secondary interventions

Promoting family bonds is identified as a highly relevant intervention during child hospitalization^(15),17,22,23)^. The kangaroo method (skin–toskin–contact) emerges as a significant intervention in reducing parental and hospitalized newborn stress^(30)^. Furthermore, this intervention appears to promote bonding and lactation, improving physiological and neurological stability, and is therefore recommended in Neonatal Intensive Care Units^(30)^. Another intervention identified as facilitating bonding is encouraging the extraction of breast milk from the newborn, which also promotes an increase in the amount of breast milk expressed^(17),22)^. It is also important to involve fathers in promoting breastfeeding, aiming at newborns’ well-being and the interaction of the triad(^17),22)^.

It is necessary to promote adaptive strategies for hospitalization, such as family coping, and simultaneously respect the decisions of parental figures, in order to promote their independence in the care provided^(18)^. Moreover, nurses should intervene with the aim of increasing parents’ self–esteem^(19)^, validating their emotions, such as remorse and guilt, and helping them to focus on the present time^(30)^ and, thus, maintain hope^(18–20 24,27)^.

The importance of encouraging and empowering parents to participate in the care provided through communication and the establishment of therapeutic relationships that promote parental empowerment is also mentioned^(29)^. They also add that, in the absence of parents, the nursing team should encourage virtual presence through the use of video calls or phone calls.

The importance of encouraging parental presence during hospitalization is also highlighted, planning and involving them in the care of the newborn and making them partners in this process^(16,17,21,22)^. In this way, parents tend to feel more involved and aware of their child’s needs, assuming the role of primary caregivers.

#### Tertiary interventions

Tertiary-level nursing interventions should ensure that, upon returning home, parents have effectively developed parenting skills, acquiring the necessary skills to meet their child’s needs and maintaining emotional bonds^(12)^.

Referrals for families and children with behavioral problems in their early stages are also highlighted^(20)^. Providing information about community and social resources to promote connection with services and thus ensure family support is also mentioned^(20)^. The use of adaptation kits, animal therapy, and therapeutic groups are mentioned as significant community resources that should be considered in nursing interventions.

### Study limitations

A limitation of this scoping review was the limited number of studies included after applying the exclusion and inclusion criteria. A concentration of studies focused on newborns was also observed, which may have limited the diversity of interventions analyzed, highlighting a gap in relation to other pediatric age groups. The review also did not analyze the effectiveness of the interventions, as its objective was mapping, not critically assessing the results. Finally, although primary, secondary, and tertiary interventions have been identified, it has not always been possible to clearly determine whether these are applicable to all hospitalization situations or whether they are restricted to specific contexts.

### Contributions to nursing

This study, by mapping knowledge about nursing interventions that promote parenting during child hospitalization in vulnerable situations, informs nursing practice, particularly in the context of child hospitalization, by offering a broad and systematic overview of interventions that can be implemented to support parenting in vulnerable situations. Primary nursing interventions that promote parenting during child hospitalization focus on health promotion and parental education. Secondary nursing interventions focus on parental empowerment, emotional support, and maintaining family bonds. Tertiary interventions, in turn, focus on connecting with community resources.

## FINAL CONSEDERATIONS

With this scope review, it was possible to map the scientific evidence regarding nursing interventions that promote parenting during child hospitalization in vulnerable situations, identifying the vulnerable situations experienced by the child and family in a hospitalization situation, as well as the needs of parents during child hospitalization in a vulnerable situation.

It is understandable that child hospitalization in a vulnerable context is a stressful time for families, often stemming from parents’ lack of understanding of how to react to a situation they’ve never experienced before. This often leads to a parental identity crisis, with a lack of clarity about their roles regarding what they can and cannot do and/or what professionals expect them to do. This is a difficult and complex situation, as nurses are responsible for boosting parents’ self-confidence so they can manage their emotions and organize themselves in the face of their child’s hospitalization.

The vulnerable situations described are mostly related to child hospitalization. Prematurity and contact with opioids in the perinatal period are also identified as situations that increase vulnerability.

The main needs of parents were identified as the need for emotional support, validation of experienced emotions, the need for a support and transportation system, and the need for health education.

Regarding nursing interventions that promote parenting during child hospitalization in vulnerable situations, they relate to parental health education, encouraging and empowering parents to participate in child care during hospitalization, and establishing therapeutic relationships.

It is concluded that the identified nursing interventions at the primary level (focusing on disease prevention and parental education), secondary level (focusing on parental training and empowerment, emotional support and support during hospitalization, and maintaining family bonds), and tertiary level (focusing on connecting with community resources to foster system reestablishment for the post-discharge period), based on a partnership care model and family-centered care, respond to parents’ needs. Thus, it becomes possible to promote parenting by encouraging the exercise of autonomy and empowering parents in vulnerable situations, such as child hospitalization, prematurity, and contact with opioids in the perinatal period.

It is considered important to carry out further studies that describe nursing interventions that promote parenting in situations of increased vulnerability, taking into account culture and different family configurations, as well as focusing on the different stages of child development throughout children’s hospitalization process.

Based on the findings mapped in this review, it is recommended that nurses adopt a holistic, family-centered approach during vulnerable child hospitalization. Interventions should range from promoting parental education to parenting skills, through emotional support, fostering family bonding, and therapeutic communication. Furthermore, it is essential that nurses encourage active parental participation in child care, including through the use of technology to maintain contact in their absence, ensuring that families are connected to community resources that can offer ongoing support after hospital discharge. Implementing these practices can significantly contribute to strengthening parenting and children’s recovery and well-being. Continuing training for nurses in this area will be important, certainly contributing to improving quality of care.

## Data Availability

The research data are available within the article.
