# Supplementary material for: Nursing interventions promoting parenting during child hospitalization: a scoping review
Source: Rev Bras Enferm. 2026 Feb 23;79:e20250103. doi: 10.1590/0034-7167-2025-0103 (PMC12933938; doi:10.1590/0034-7167-2025-0103)
Supplement: Translation

# Translation: Intervenções de enfermagem promotoras da parentalidadedurante a hospitalização da criança: revisão de escopo

**DOI:** 10.1590/0034-7167-2025-0103pt

**Authors:** Joana Rita Guarda da Venda Rodrigues, Bárbara Rodrigues e Sousa, Francisca Cardoso Macedo, Mariana Fidalgo Cabouco, Mariana Marreiros da Rocha, Tatiana Filipa Tiago Carneiro

**Affiliations:** I Escola Superior de Enfermagem de Lisboa. Lisboa, Portugal; II Centro de Investigação, Inovação e Desenvolvimento em Enfermagem de Lisboa. Lisboa, Portugal; III Unidade Local de Saúde Lisboa Ocidental, Hospital de S. Francisco Xavier. Lisboa, Portugal; IV Unidade Local de Saúde Arco Ribeirinho, Hospital de Nossa Senhora do Rosário. Barreiro, Portugal

**Keywords:** Parentalidade, Enfermagem Pediátrica, Família, Criança, Hospitalização

## Abstract

**Objetivos:**

mapear o conhecimento sobre as intervenções de enfermagem promotoras da parentalidade durante a hospitalização da criança.

**Métodos:**

revisão de escopo, realizadaconforme metodologia do *JBI* e cordo com ochecklist Preferred Reporting Items for Systematic reviews and Metanalyses extension for Scoping Reviews.

**Resultados:**

foram identificadas 1.491 publicações, sendo incluídos 17 estudos Evidenciou–se que as intervenções de enfermagem primárias, promotoras da hospitalização da criança, estão focadas na promoção dasaúde e na educação parental. As intervenções de enfermagem secundárias se concentram no empoderamento parental, no apoio emocional e na manutenção do vínculo familiar. E as intervenções terciárias incidem na ligação com os recursos comunitários.

**Considerações Finais:**

as intervenções de enfermagem mapeadas, fundamentadas em um modelo de parceria no cuidado e na abordagem centrada na família, demonstram potencial para responder às necessidades dos pais e promover a parentalidade durante o processo de hospitalização da criança em condição de vulnerabilidade acrescida.

## INTRODUÇÃO

A parentalidade constitui uma componente essencial ao bom desenvolvimento da criança, permitindo a vinculação segura ^(1)^. A parentalidade é definida pelo *Internacional Council of Nurses* como tomar conta, em que os pais assumem as responsabilidades de ser mãe/pai, possuem comportamentos destinados a facilitar a incorporação de um recém–nascido na unidade familiar, de forma a otimizar o crescimento e desenvolvimento das crianças, interiorizando as expectativas dos indivíduos, famílias, amigos e sociedade quanto aos comportamentos de papel parental adequados ou inadequados^(2)^.

A hospitalização está amplamente retratada na literatura como um momento de estresse para as crianças e para a sua família^(3,3)^, seja por confirmação de um diagnóstico ou por agudização de uma situação clínica, consubstanciando–se, assim, pela possibilidade da ocorrência de eventos adversos, em situação de vulnerabilidade^(4^) acrescida, tanto para a criança quanto para a sua família.

Neste contexto, a vulnerabilidade, compreendida como um fenômeno dinâmico e processual^(5^) associado à fragilidade ou ao risco de sofrer danos, frequentemente presente em situações de exposição a condições ou experiências adversas multicausais e intersetoriais, que representam uma ameaça ao desenvolvimento infantil^(6^), pode ser atenuada por intervenções centradas na família^(6^), com enfoque na promoção de competências parentais^(5^).

Para Betty Neuman, as intervenções de enfermagem são definidas como ações intencionais que auxiliam o cliente a manter ou atingir a estabilidade do seu sistema^(7^). A autora identifica três níveis de intervenções: primárias, secundárias e terciárias^(7^). A intervenção primária é desenvolvida no momento em que há uma suspeita de estressor, isto é, ainda não ocorreu nenhuma reação, porém o risco de se concretizar é conhecido^(7^). Por outro lado, a intervenção secundária já “(...) envolve intervenções ou tratamento iniciados após a ocorrência de sintomas de stress”, na tentativa de se estabilizar o sistema^(7^). Por fim, as intervenções terciárias ocorrem após o tratamento ativo, centrando–se no reajustamento do sistema, com o objetivo de voltar a adquirir a estabilidade^(7^).

As intervenções de enfermagem no contexto de hospitalização da criança e da sua família podem potenciar a autoconfiança parental, favorecendo a regulação emocional e a reorganização diante do novo cenário de hospitalização do filho^(8^). Ao criar oportunidades que evidenciem as competências dos pais, essas intervenções contribuem para aquisição de novas capacidades, que permitem aos pais responder de forma mais adequada às necessidades dos filhos^(7^). Neste contexto de hospitalização, os cuidados centrados na família e a parceria de cuidados surgem como pilares centrais de intervenção^(9^). A filosofia de cuidados centrados na família defende o papel integral desta na vida da criança, reconhecendo–a como elemento essencial e integrante dos cuidados e identificando a capacitação e o empoderamento como conceitos fundamentais^(10^). O *Institute for Patient–and FamilyBCentered Care* acrescenta que o respeito e a dignidade, a partilha de informação, a participação e a colaboração constituem conceitos essenciais para o desenvolvimento de uma prática sustentada nos cuidados centrados na família^(11)^. Por sua vez, o modelo de parceria nos cuidados desenvolvido por Anne Casey, em 1988, assenta no reconhecimento e no respeito pelo papel da família no cuidado à criança^(12^).

Existem múltiplos estudos sobre a hospitalização da criança, sobretudo com enfoque no recém–nascido prematuro hospitalizado em uma Unidade de Terapia Intensiva Neonatal^(1)^. A revisão escopo desenvolvida por Barros *et al*.^(13^) foca apenas nas intervenções de enfermagem promotoras da adaptação da criança/jovem/família à hospitalização. Por sua vez, a revisão desenvolvida por Querido *et al*.^(1)^ apenas incide nas intervenções de enfermagem promotoras da vinculação ao recém–nascido hospitalizado, tendo–se verificado que as situações de vulnerabilidade descritas se relacionam, na maioria das vezes, com a hospitalização da criança. A prematuridade e o contato com opioides no período perinatal são identificados, ainda, como situações que acrescem vulnerabilidade.

Esta revisão, por sua vez, centra–se tanto nos aspectos relacionados à parentalidade no contexto hospitalar quanto no fortalecimento do vínculo e das competências parentais, independentemente da idade da criança ou da razão da hospitalização, o que não foi explorado nas revisões anteriormente referidas. Também a partir da pesquisa preliminar da literatura efetuada nas bases de dados JBI, *Prospero e Open Science Framework* (OSF) em março de 2024, não foram identificadas revisões com enfoque nas intervenções de enfermagem promotoras da parentalidade durante a hospitalização da criança em situação de vulnerabilidade, verificando–se, concomitantemente, que a literatura sobre o fenômeno em estudo se encontrava dispersa. Assim, identificar a evidência científica disponível permitirá sintetizar o conhecimento sobre as intervenções de enfermagem que promovem a parentalidade na hospitalização da criança, orientando a prática e sinalizando lacunas para futuras investigações.

Na convergência do exposto, a presente revisão tem como objetivo mapear o conhecimento sobre as intervenções de enfermagem promotoras da parentalidade durante a hospitalização da criança, dando resposta à questão “Quais são as intervenções de enfermagem que promovem o exercício da parentalidade durante a hospitalização da criança?” e às subquestões “Quais as situações de vulnerabilidade vivenciadas pelos pais e família durante a hospitalização?” e “Quais as necessidades dos pais e da família durante a hospitalização da criança?”.

## OBJETIVOS

Mapear o conhecimento sobre as intervenções de enfermagem promotoras da parentalidade durante a hospitalização da criança.

## MÉTODOS

A presente revisão de escopo foi realizada conforme a metodologia recomendada pelo JBI e de acordo com *Preferred Reporting Items for Systematic reviews and Meta–Analyses extension for Scoping Reviews* (PRISMA–ScR)^(14^).

### Critérios de elegibilidade

Os critérios de elegibilidade dos estudos selecionados foram definidos com base na mnemônica PCC (População, Conceito e Contexto). Assim, a População (P) desta revisão de escopo integra os pais e a família de crianças hospitalizadas que se encontram em situação de vulnerabilidade; o Conceito (C) corresponde às intervenções de enfermagem promotoras da parentalidade em situação de vulnerabilidade; e o Contexto (C) é a hospitalização da criança.

Foram incluídos na presente revisão estudos primários que abordavam intervenções de enfermagem promotoras da parentalidade durante a hospitalização da criança. Foram excluídas as produções que incidiam apenas em intervenções de enfermagem focadas na vinculação ao recém–nascido hospitalizado. Não foi estabelecido limite temporal. O protocolo foi redigido, aferido e revisto pelos autores, tendo sido registado prospectivamente na OSF (https://osf.io/vjdb5/).

### Estratégia de pesquisa e identificação dos estudos

Para identificação dos estudos, foram utilizadas as bases de dados eletrônicas MEDLINE (via PubMed), CINAHL *Unlimited* (via EBSCO), Scopus, *Psychology Behavioral Science Collection*, MedicLatina, *Web of Science, Cochrane Central Register of Controlled Trials, Cochrane Database of Systematic Reviews, Cochrane Clinical Answers* e ERIC. Para identificar os estudos não publicados, foi realizada a pesquisa nos Repositórios Científicos de Acesso Aberto de Portugal (RCAAP), *Google Scholar e OpenAIRE*.

A pesquisa teve início no dia 20 de março de 2024, com a pesquisa nas bases de dados eletrônicas MEDLINE (via PubMed) e CINAHL *Unlimited* (via EBSCO). Foram mobilizados termos de pesquisa em linguagem natural, de forma a auxiliar a identificação das palavras–chave utilizadas nos títulos e resumos, tal como os termos de indexação. Posteriormente, as palavras naturais, as palavras–chave e os termos de indexação listados foram combinados com operadores booleanos e, quando possível, um operador asterisco (*), de modo a formar a expressão de pesquisa (TI Parent* OR TI Family OR AB Parent* OR AB Family) AND (TI Pediatric* OR TI Nurs* OR TI Neonatal* OR TI Hospital* OR AB Pediatric* OR AB Nurs* OR AB Neonatal* OR AB Hospital*) AND (TI Vulnerability OR TI “Special Populations” OR AB Vulnerability OR AB “Special Populations”)), que foi adaptada às especificidades de cada base de dados ou repositório.

Em seguida, analisaram–se as referências bibliográficas nos registros anteriormente identificados. Foram, também, procurados estudos não publicados e literatura cinzenta no RCAAP, *Google Scholar* e *OpenAIRE*.

### Extração, análise e síntese dos dados

Os resultados da pesquisa foram exportados para o gestor de referências *Mendeley Desktop* (*version* 1.19.8), através do qual foram identificados e removidos os registros duplicados. Em seguida, para auxiliar a seleção dos artigos, os registros foram introduzidos na plataforma *Qatar Computing Research Institute* (Rayyan QCRI). A elegibilidade dos documentos foi apurada por dois revisores de forma independente e cega, através da avaliação dos títulos e resumos relativos aos critérios de inclusão e exclusão para revisão, recorrendo–se a um terceiro revisor para clarificar divergências apontadas. Os artigos que cumpriram os critérios de elegibilidade foram recuperados na íntegra, e o texto completo foi avaliado detalhadamente em relação à sua elegibilidade por dois ou mais revisores de forma independente e cega. Sempre que o texto integral não estava disponível, foi contatado o autor correspondente e/ou solicitada a obtenção do artigo à biblioteca de uma universidade.

A análise descritiva e síntese dos dados dos registros que cumpriram os critérios de inclusão e exclusão foram realizadas, de forma independente e cega, por dois ou mais autores, com recurso a quadros desenvolvidos pelos próprios. Estes incluíram dimensões de caracterização das publicações e dos estudos selecionados, bem como da situação de vulnerabilidade, das necessidades parentais e das intervenções de enfermagem, em consonância com os objetivos e a questão de revisão de escopo. Após a revisão mútua dos registros, os autores identificaram e assinalaram as áreas de incerteza. As dúvidas foram resolvidas por meio de discussão, recorrendo–se a um terceiro revisor para clarificar divergências, culminando na apresentação dos resultados consensualizados.

## RESULTADOS

A pesquisa realizada nas bases de dados permitiu identificar 1.491 publicações, tendo sido incluídos 17 artigos. Os resultados obtidos nas diferentes etapas de seleção dos estudos são apresentados de acordo com as recomendações do PRISMA–ScR^(12^), explanado na Figura 1.

**Figure 1:** Preferred Reporting Items for Systematic reviews and Meta–Analyses extension for Scoping Reviews (adapted) flowchart of the study selection process, Lisbon, Portugal

A partir do processo de seleção realizado, resultaram 17 estudos, que foram integrados na presente revisão de escopo. Os resultados encontram–se resumidos e organizados nos Quadros 1 e 2, e na Figura 2, respetivamente.

**Quadro 1:** Caracterização geral dos estudos incluídos na revisão de escopo, Lisboa, Portugal

Nº do estudo (E) Título | autor(es)	País | ano	Tipo de estudo	Objetivo(s)	Participantes | caracterização
E1 Parent Visiting and Participation in Infant Caregiving Activities in a Neonatal Unit Franck *et al*.^(15^	Reino Unido, 2003	Qualitativo	Descrever a frequência e duração da visita dos pais, participação nos cuidados e identificar fatores associados à participação dos pais.	110 pais que visitam os seus bebês em uma unidade neonatal.
E2 Adjusting to being a father to an infant born prematurely: experiences from Swedish fathers Lindberg *et al*.^(16)^	Suécia, 2008	Qualitativo	Descrever as experiências de ser pai de um recém–nascido prematuro.	Oito pais de bebês prematuros que foram cuidados em uma Unidade de Terapia Intensiva Neonatal no Norte da Suécia.
E3 The parent–nurse relationship in the neonatal intensive care unit context – closeness and emotional involvement Fegran *et al*.^(17)^	Noruega, 2009	Qualitativo	Explorar as experiências dos pais e enfermeiros sobre a relação entre ambos quando o filho prematuro é hospitalizado.	Seis mães, seis pais e seis enfermeiros em uma Unidade de Terapia Intensiva Neonatal na Noruega.
E4 Percepções da família acerca das dificuldades de adaptação da criança à hospitalização: subsídios para a enfermagem Gomes *et al*.^(18)^	Brasil, 2013	Qualitativo exploratório– descritivo	Conhecer as percepções da família acerca das dificuldades de adaptação da criança durante a hospitalização.	15 familiares de três crianças internadas.
E5 Validation of Hospitalization Impact Scale among families with children hospitalized for cancer treatment Lyu *et al*.^(19)^	China, 2015	Quantitativa e qualitativa	Modificar e validar as propriedades psicométricas da *Hospitalization Impact Scale* para avaliar o impacto da hospitalização de crianças com cancro nas suas famílias.	253 famílias com crianças hospitalizadas para tratamento de cancro em quatro departamentos de oncologia pediátrica em hospitais da China.
E6 Interactions Between Children And Pediatric Nurses At The Emergency Department: A Swedish Interview Study Grahn *et al*.^(20)^	Suécia, 2016	Qualitativo	Descrever estratégias utilizadas pelos enfermeiros na interação com crianças dos 3 aos 6 anos.	Sete enfermeiros com experiência em cuidados pediátricos.
E7 Differences And Similarities Between Mothers And Fathers Of Premature Children: A Qualitative Study Of Parents’ Coping Experiences In A Neonatal Intensive Care Unit Hagen *et al*.^(21)^	Noruega, 2016	Qualitativo	Explorar e descrever as experiências de *coping* dos pais de crianças admitidas em uma Unidade de Terapia Intensiva Neonatal.	Oito mães e oito pais que foram entrevistados entre um a seis meses após a alta da Unidade de Terapia Intensiva Neonatal.
E8 A Life Uncertain – My Baby’s Vulnerability: Mothers’ Lived Experience Of Connection With Their Preterm Infants In A Botswana Neonatal Intensive Care Unit Ncube *et al*.^(22)^	Botsuana, 2016	Qualitativo	Explorar e descrever as experiências vividas por mães em relação ao cuidado de bebês prematuros hospitalizados em uma Unidade de Terapia Intensiva Neonatal.	Oito mães de bebês prematuros que foram admitidos em uma unidade neonatal imediatamente após o parto.
E9 Supporting Of The Fathers To Visit Their Infants In Neonatal Intensive Care Unit Decreases Their Stress Level: A Pretest– Posttest Quasi–Experimental Study Özdemir & Alemdar^(23)^	Turquia, 2016	Quase– experimental	Determinar o efeito do apoio e das visitas dos pais aos seus bebês na Unidade de Terapia Intensiva Neonatal nos níveis de estresse dos pais.	47 pais de bebês internados em uma Unidade de Terapia Intensiva Neonatal em um Hospital da Turquia Oriental.
E10 The Lived Experience of Jordanian Parents In a Neonatal Intensive Care Unit: A Phenomenological Study Abuidhail *et al*.^(24)^	Jordânia, 2017	Qualitativo	Descrever as experiências vividas, as necessidades em relação ao cuidado e os sistemas de apoio dos pais de neonatos admitidos em uma Unidade de Terapia Intensiva Neonatal.	Dez participantes, sendo oito mães e dois pais de neonatos em Unidade de Terapia Intensiva Neonatal.
E11 Experiences and needs of parents of critically injured children during the acute hospital phase: A qualitative investigation Foster *et al*.^(25)^	Austrália, 2017	Qualitativo, parte de estudo longitudinal misto	Explorar as experiências dos pais de crianças criticamente feridas durante a fase aguda de hospitalização e determinar as suas necessidades de apoio durante esse período.	40 pais de 30 crianças criticamente feridas, com idades entre 1 e 13 anos.
E12 Family Stress in Pediatric Critical Care Hagstrom^(26)^	Estados Unidos da América, 2017	Qualitativo e quantitativo	Descrever as fontes de estresse para famílias cujas crianças foram hospitalizadas na Unidade de Terapia Intensiva Pediátrica por mais de uma semana.	Nove pais (oito mães e um pai) de oito famílias, todos casados.
E13 Sistemas de apoio na unidade de terapia intensiva pediátrica: perspectiva dos familiares Bazzan *et al*.^(27)^	Brasil, 2019	Qualitativo	Identificar e analisar os sistemas de apoio utilizados pelos familiares para o processo de adaptação à hospitalização da criança na Unidade de Terapia Intensiva Pediátrica.	13 familiares de crianças internadas na Unidade de Terapia Intensiva Pediátrica.
E14 Engaging Mothers to Implement Nonpharmacological Care for Infants with Neonatal Abstinence Syndrome: Perceptions of Perinatal and Pediatric Nurses Shuman *et al*.^(28)^	Estados Unidos da América, 2020	Qualitativo	Descrever a percepção de enfermeiros pediátricos e perinatais sobre a participação de mães nos cuidados a bebês expostos a opioides, bem como os fatores facilitadores e barreiras à participação.	21 enfermeiros do bloco de partos, da Unidade de Terapia Intensiva Neonatal e do serviço de pediatria.
E15 Parental presence, participation, and engagement in paediatric hospital care: A conceptual delineation Harlow *et al*.^(29)^	Estados Unidos da América, 2023	Análise conceptual	Delinear os conceitos sobre a presença parental, participação e *engagement* nos cuidados hospitalares pediátricos.	Publicações entre 1991—2003.
E16 O exercício parental durante a hospitalização do filho: modelo de intencionalidades terapêuticas de enfermagem face à parceria de cuidados Sousa *et al*.^(12)^	Portugal, 2023	Investigação– ação	Identificar as intencionalidades terapêuticas dos enfermeiros quando promovem a parceria de cuidados com os pais durante a hospitalização da criança.	Enfermeiros do serviço de internamento de pediatria do Hospital Pedro Hispano, os pais e as crianças hospitalizadas nesse serviço.
E17 Mother’s mental health and the interaction with her moderate preterm baby in the NICU Mira *et al*.^(30)^	Chile, 2024	Correlacional	Descrever o impacto de ter um recém–nascido de pré–termo moderado hospitalizado em uma Unidade de Terapia Intensiva Neonatal na saúde mental das mães e sua relação com a interação da díade.	85 díades mãe–recém–nascido de pré–termo moderado.

**Quadro 2:** Descrição das situações de vulnerabilidade, das necessidades parentais e intervenções de enfermagem que promovem o desempenho da parentalidade durante a hospitalização da criança, Lisboa, Portugal

Nº do estudo Autor(es) Ano	Situação de vulnerabilidade	Necessidades parentais e/ou familiares	Intervenções de enfermagem
E1 Franck *et al*. (2003)^(15)^	Hospitalização da criança Prematuridade	Os pais, em particular as mães, necessitam de apoio emocional e prático durante a hospitalização do bebê, bem como de participar ativamente nos cuidados ao recém–nascido, de forma a favorecer o vínculo afetivo e reduzir o estresse.	Incentivar a participação dos pais nos cuidados aos filhos; Promover a proximidade física; Promover a amamentação; Apoiar emocional aos pais; Comunicar com os pais; Participação nos cuidados de rotina.
E2 Lindberg *et al*. (2008)^(16)^	Hospitalização da criança Prematuridade	Os pais de recém–nascidos prematuros expressaram a necessidade de apoio emocional e prático, incluindo a educação sobre os cuidados com o bebê e validação de emoções como culpa e ansiedade.	Incentivar a participação dos pais nos cuidados aos filhos; Promover a amamentação; Estabelecer uma relação de parceria; Fornecer apoio emocional; Acompanhar na primeira visita ao recém–nascido; Promover a proximidade, o contato, o toque, calor e odor através da voz; Promover o contato pele a pele através do método canguru; Planear, com os pais, a prestação de cuidados.
E3 Fegran *et al*. (2009)^(17)^	Hospitalização da criança	Os pais expressam a necessidade de apoio emocional e de respeito enquanto parceiros no cuidado dos filhos, manifestando igualmente o desejo de serem compreendidos e de verem as suas emoções validadas.	Incentivar a participação dos pais nos cuidados aos filhos; Promover a proximidade física entre os pais e os recém– nascidos através do toque, contato pele–a–pele e massagem; Consolidar as capacidades parentais e o desenvolvimento social e psicológico; Estabelecer uma boa comunicação, fornecendo todas as informações relativas ao recém–nascido nas diferentes fases do internamento; Fornecer apoio emocional; Promover o método canguru; Incentivar as mães a extrair leite junto dos recém–nascidos; Envolver os pais nos cuidados ao recém–nascido.
E4 Gomes *et al*. (2013)^(18)^	Hospitalização da criança	As famílias necessitam de apoio emocional e prático para apoiar a criança na adaptação à hospitalização.	Comunicar efetivamente com a família; Educar sobre as capacidades parentais e conhecimentos sobre os cuidados à criança; Promover relaxamento ou distração através da realização de atividades; Encorajar a expressão emocional adaptativa; Ajudar os pais a identificar recursos de apoio social.
E5 Lyu *et al*. (2015)^(19)^	Hospitalização da criança	Os pais necessitam de apoio emocional e prático, incluindo na identificação de estressores específicos durante a hospitalização, e de intervenções direcionadas às suas necessidades, como a identificação de estressores específicos durante a hospitalização e a procura de intervenções que atendam às suas necessidades.	Comunicar efetivamente com a família; Educar sobre as capacidades parentais e conhecimentos sobre os cuidados à criança; Ajudar os pais a identificar recursos de apoio social.
E6 Grahn *et al*. (2016)^(20)^	Hospitalização da criança	Necessidade de interações entre enfermeiros e crianças no departamento de emergência que promovam um ambiente seguro e acolhedor, valorizando a comunicação e a participação dos pais para reduzir a ansiedade e melhorar a experiência do cuidado.	Comunicar efetivamente com a família; Educar sobre as capacidades parentais; Transmitir conhecimentos sobre os cuidados à criança; Promover relaxamento ou distração através da realização de atividades; Ensinar estratégias de controle da ansiedade e do estresse aos pais; Encorajar a expressão emocional adaptativa; Ajudar os pais a identificar recursos de apoio social.
E7 Hagen *et al*. (2016)^(21)^	Hospitalização da criança Prematuridade	Os pais expressaram a necessidade de apoio emocional e informações claras sobre o estado de saúde dos filhos. Relataram a importância de serem ouvidos em relação aos cuidados com o recém– nascido prematuro e de ter a presença do parceiro para melhorar o *coping*.	Incentivar a participação dos pais nos cuidados aos filhos; Promover a proximidade física entre os pais e os recém– nascidos através do toque, contato pele–a–pele e massagem; Comunicar com os pais; Promover apoio emocional aos pais; Promover a participação dos nos cuidados de rotina.
E8 Ncube *et al*. (2016)^(22)^	Hospitalização da criança Prematuridade	As mães de recém–nascidos prematuros necessitam de apoio emocional, informações claras sobre o estado de saúde do filho e oportunidades para participar ativamente nos cuidados.	Incentivar a participação dos pais nos cuidados aos filhos; Promover a proximidade física entre os pais e os recém– nascidos através do toque, contato pele–a–pele e massagem; Promover a amamentação; Consolidar as capacidades parentais e o desenvolvimento social e psicológico; Estabelecer uma relação de parceria; Estabelecer uma boa comunicação, fornecendo todas as informações relativas ao recém–nascido nas diferentes fases do internamento; Acompanhar na primeira visita ao recém–nascido; Promover o método canguru; Incentivar as mães a extrair leite junto do recém–nascido; Planear, com os pais, a prestação de cuidados.
E9 Özdemir & Alemdar (2016)^(23)^	Hospitalização da criança Prematuridade	Os pais necessitam de apoio emocional e informações claras sobre a condição do filho como estratégia para reduzir o estresse e promover a adaptação.	Incentivar a participação dos pais nos cuidados aos filhos; Promover a proximidade física; Comunicar com os pais; Promover apoio emocional aos pais; Promover a participação dos pais nos cuidados de rotina.
E10 Abuidhail et al. (2017)^(24)^	Hospitalização da criança	Os pais referem necessidade de informações e intervenção dos enfermeiros para enfrentar situações de estresse, bem como de apoio emocional e psicológico.	Comunicar efetivamente com a família; Educar sobre as capacidades parentais e conhecimentos sobre os cuidados à criança; Capacitar os pais para gerir a ansiedade e o estresse; Encorajar a expressão emocional adaptativa.
E11 Foster *et al*. (2017)^(25)^	Hospitalização da criança	Os pais necessitam de apoio psicológico e informações claras sobre o tratamento e a recuperação dos filhos, manifestando também preocupações com o impacto emocional das lesões nas crianças e na dinâmica familiar.	Comunicar efetivamente com a família; Educar sobre as capacidades parentais e conhecimentos sobre os cuidados à criança; Ajudar os pais a identificar recursos de apoio social.
E12 Hagstrom (2017)^(26)^	Hospitalização da criança	Os pais expressam a necessidade de apoio emocional e prático, incluindo a validação de emoções, como a culpa e o remorso, e a necessidade de manter a conexão com os filhos que estão em casa. Além disso, sentem necessidade de informações claras e honestas sobre a condição de saúde dos seus filhos e sobre o que esperar durante a hospitalização.	Comunicar efetivamente entre os profissionais de saúde e a família; Educar sobre as capacidades parentais e conhecimentos sobre os cuidados à criança; Promover relaxamento ou distração através da realização de atividades; Capacitar os pais para gerir a ansiedade e o estresse; Encorajar a expressão emocional adaptativa; Ajudar os pais a identificar recursos de apoio social.
E13 Bazzan *et al*. (2019)^(27)^	Hospitalização da criança	As famílias necessitam de um sistema de apoio robusto que inclua suporte emocional de familiares, amigos e profissionais de saúde, além de interações com outros familiares de crianças internadas.	Comunicar efetivamente com a família; Educar sobre as capacidades parentais e conhecimentos sobre os cuidados à criança; Ajudar os pais a identificar recursos de apoio social.
E14 Shuman *et al*. (2020)^(28)^	Contato com opioides no período perinatal	Ajuda nos cuidados ao recém–nascido por viver longe, não ter transporte, existir limites no tempo de visita ou ter outros filhos.	Educar antes do parto; Validar as emoções das mães (remorso e culpa); Ajudar as mães a se focar no que podem fazer no momento presente; Comunicar objetiva e honestamente sobre o seu histórico de consumo de substâncias e os sintomas de abstinência do bebê.
E15 Harlow *et al*. (2023)^(29)^	Hospitalização da criança	Estar fisicamente presente para acompanhar os filhos durante o internamento.	Estimular a presença física dos pais e, se não for possível, recorrer à presença virtual através de videochamadas ou chamadas telefônicas; Incentivar a participação dos pais nos cuidados aos filhos, nomeadamente na alimentação, troca de fralda e promoção do conforto; Empoderar os pais a participar nos cuidados através do estabelecimento de uma relação que promova a educação dos pais e a sua participação; Incentivar a técnica do canguru e a presença junto aos filhos.
E16 Sousa *et al*. (2023)^(12)^	Hospitalização da criança	Envolvimento, ou não, no processo de cuidados, de acordo com as necessidades dos pais. Estabelecimento de relações terapêuticas com os enfermeiros.	Promover a participação dos pais nos cuidados promotores do desenvolvimento; Promover competências parentais para prestar cuidados complexos; Reduzir o nível de desgaste dos pais de crianças com necessidades especiais permanentes; Preparar os pais para promover a autonomia da criança.
E17 Mira *et al*. (2024)^(30)^	Hospitalização da criança Prematuridade	Necessidade de educação e apoio durante a hospitalização, assim como acesso sem limitações à Unidade de Terapia Intensiva Neonatal.	Facilitar o contato pele a pele precoce e frequente durante, pelo menos, 60 minutos para reduzir o estresse parental e da criança; Promover a amamentação.

**Figure 2:** Preferred Reporting Items for Systematic reviews and Meta–Analyses extension for Scoping Reviews (adapted) flowchart of the study selection process, Lisbon, Portugal

Os 17 estudos incluídos na presente revisão de escopo foram realizados entre 2003 e 2024 em 12 países diferentes, nomeadamente: Estados Unidos da América ^(3^); Brasil ^(2^); Noruega ^(2^); Suécia ^(2^); Chile ^(1)^; Portugal ^(1)^; Austrália ^(1)^; China ^(1)^; Jordânia ^(1)^; Botsuana ^(1)^; Turquia ^(1)^; Reino Unido ^(1)^. No que se refere ao desenho dos estudos, identificam–se 11 estudos qualitativos, dois estudos mistos, um estudo correlacional, um estudo de investigação–ação, um estudo quase–experimental e uma análise conceptual.

Da revisão resultante dos artigos selecionados e analisados, emergiram informações pertinentes relativas às: i) situações de vulnerabilidade da criança; ii) necessidades dos pais; iii) intervenções de enfermagem promotoras da parentalidade durante a hospitalização da criança em situação de vulnerabilidade, que dão resposta aos objetivos inicialmente estabelecidos, conforme pode ser consultado no Quadro 2 e Figura 2.

## DISCUSSÃO

A parentalidade pode ser entendida como o assumir das responsabilidades de ser pai de uma criança durante o seu desenvolvimento^(12^). Um processo de hospitalização de uma criança representa um evento crítico e uma situação de vulnerabilidade no exercício da parentalidade^(12^). Durante o internamento dos filhos, os pais não experienciam apenas o estresse do ambiente, mas também complicações ou incertezas em relação ao estado de saúde da criança, à separação física e emocional do seu bebê, e a níveis acrescidos de estresse parental^(30^). De acordo com o estudo desenvolvido por Mira, Coo e Bastías, que teve como objetivo descrever o impacto do internamento de recém–nascidos prematuros na saúde mental materna, cerca de 92% das mães relataram que a hospitalização na Unidade de Terapia Intensiva Neonatal constituiu uma experiência geradora de estresse, sendo o exercício da parentalidade a principal fonte de estresse^(30^).

A atitude dos pais devido à hospitalização do filho depende das características pessoais da criança, dos próprios pais e dos enfermeiros, pois o estabelecimento de uma parceria de cuidados entre enfermeiros e pais se configura como um caminho para alcançar melhores resultados^(12^). Assim, a capacidade dos enfermeiros para intervir em parceria com os pais constitui uma competência essencial para atingir padrões mais elevados de qualidade nos cuidados de enfermagem prestados^(12)^.

### Situações de vulnerabilidade vivenciadas pela criança e família

As situações de vulnerabilidade em contexto hospitalar identificadas nos estudos incluídos estão, na sua maioria, relacionadas à própria hospitalização da criança^(18–20,24–27)^. O contexto hospitalar parece alterar a capacidade de adaptação da criança, aumentando a sua vulnerabilidade^(18–20,24–27)^. A prematuridade foi, também, identificada como uma situação de vulnerabilidade, uma vez que os recém–nascidos pré–termo apresentam um maior risco de desenvolver problemas a nível neurocomportamental, quando comparados com os recém–nascidos a termo^(30)^. Para além das situações anteriormente referidas, o contato com opioides, no período perinatal, também foi identificado como uma situação de vulnerabilidade vivida pela criança^(28)^. 

### Necessidades dos pais durante a hospitalização da criança em situação de vulnerabilidade

A análise dos estudos selecionados evidenciou diversas necessidades dos pais durante a hospitalização dos seus filhos. Alguns autores se centram na necessidade de educação em saúde, nomeadamente no que diz respeito à amamentação, ao desenvolvimento e aos cuidados à criança, bem como à gestão da ansiedade, do estresse e dos sintomas associados à síndrome de abstinência^(30)^. É ainda salientada a importância do estabelecimento de uma relação de parceria com os profissionais de saúde, nomeadamente com os enfermeiros, na prestação de cuidados aos filhos durante a hospitalização^(18–20,25,26)^.

Igualmente, os estudos identificados mostram que muitos pais têm dificuldade de acesso aos serviços, seja porque moram longe ou porque não têm acesso a transporte, o que pode comprometer o estabelecimento de uma relação terapêutica com as equipes de saúde e a participação nos cuidados aos filhos^(28)^. Os pais manifestam, ainda, a necessidade de verem validadas as suas emoções pelos enfermeiros, de modo a consolidar a relação terapêutica em uma base empática^(18,19,25,26)^.

### Intervenções de enfermagem promotoras da parentalidade durante a hospitalização da criança em situação de vulnerabilidade 

#### Intervenções primárias

As intervenções de enfermagem primárias centram-se na promoção da parentalidade, reforçando o exercício parental durante a hospitalização, garantindo a sua continuidade e preparando os pais para fomentar a autonomia da criança^(12)^. Entre as intervenções primárias identificadas, destacam–se a educação parental^(18,19,24–27)^ e a educação em saúde no período pré–parto, nomeadamente no que respeita ao consumo de substâncias pela mãe e aos sintomas de abstinência do recém–nascido^(28)^.

#### Intervenções secundárias

A promoção do vínculo familiar é identificada como uma intervenção de grande relevância durante a hospitalização da criança^(15,17,22,23)^. O método canguru (contato pele–a–pele) emerge como uma intervenção significativa na redução do estresse parental e do recém–nascido hospitalizado^(30)^. Além disso, esta intervenção parece favorecer a vinculação e lactação, melhorando a estabilidade fisiológica e neurológica, sendo, por isso, recomendada nas Unidades de Terapia Intensiva Neonatal^(30)^. Outra intervenção identificada como facilitadora da vinculação consiste em incentivar a extração de leite materno junto ao recém–nascido, o que também promove o aumento da quantidade de leite materno extraído^(17,22)^. Destaca–se, ainda, a importância de envolver o pai na promoção da amamentação, visando ao bem–estar do recém–nascido e à interação da tríade^(17,22)^.

É necessário promover estratégias adaptativas à hospitalização, como o *coping familiar* e, simultaneamente, respeitar as decisões das figuras parentais, de maneira a promover a sua independência nos cuidados prestados^(18)^. Além disso, os enfermeiros devem intervir com o objetivo de aumentar a autoestima dos pais^(19)^, validando as emoções dos mesmos, como o remorso e a culpa, e auxiliando–os a focar–se no tempo presente^(30)^ e, dessa forma, manter a esperança^(18–20,24–27)^.

É referida, ainda, a importância de encorajar e empoderar os pais a participar nos cuidados prestados, através da comunicação e do estabelecimento de relações terapêuticas que promovam o *empowerment parental* ^(29)^. Acrescentam ainda que, na ausência dos pais, a equipe de enfermagem deve incentivar a presença virtual através do recurso a videochamadas ou chamadas telefônicas^(29)^.

É igualmente salientada a importância de incentivar a presença dos pais durante a hospitalização, planeando e envolvendo–os na prestação de cuidados ao recém–nascido e tornando–os parceiros nesse processo^(16,17,21,22)^. Dessa forma, os pais tendem a se sentir mais envolvidos e conscientes das necessidades do filho, assumindo o papel de principais cuidadores. 

#### Intervenções terciárias

As intervenções de enfermagem de nível terciário devem assegurar que, no regresso à casa, os pais tenham desenvolvido, de forma eficaz, o exercício da parentalidade, adquirindo as competências necessárias para responder às necessidades do filho e preservando os laços afetivos^(12)^. 

A referenciação de famílias e crianças com problemas de comportamento, na sua fase inicial, também é evidenciada^(20)^. O fornecimento de informações sobre os recursos da comunidade e sociais, de forma a promover a ligação com os serviços e, assim, assegurar o acompanhamento da família, também é enunciado^(20)^. A utilização de *kits* de adaptação, da terapia com animais e de grupos terapêuticos é referida como recurso comunitário significativo que deve ser contemplado nas intervenções de enfermagem.

### Limitações do estudo

Foi evidenciada como limitação a esta revisão de escopo a existência de um número reduzido de estudos incluídos após terem sido aplicados os critérios de exclusão e inclusão. Observou–se ainda uma concentração de estudos com enfoque no recém–nascido, o que pode ter restringido a diversidade das intervenções analisadas, evidenciando uma lacuna em relação a outras faixas etárias pediátricas. A revisão também não analisou a efetividade das intervenções, uma vez que seu objetivo foi de mapeamento, não de avaliação crítica dos resultados. Por fim, embora tenham sido identificadas intervenções primárias, secundárias e terciárias, nem sempre foi possível determinar com clareza se essas são aplicáveis a todas as situações de hospitalização ou se estão restritas a contextos específicos.

### Contribuições para a área da enfermagem

Este estudo, ao mapear o conhecimento sobre as intervenções de enfermagem promotoras da parentalidade durante a hospitalização da criança em situação de vulnerabilidade, informa sobre a prática de enfermagem, principalmente no contexto da hospitalização infantil, ao oferecer uma visão ampla e sistemática das intervenções que podem ser implementadas para apoiar a parentalidade em situações de vulnerabilidade. As intervenções de enfermagem primárias promotoras da parentalidade durante a hospitalização da criança focam na promoção da saúde e na educação parental. As intervenções de enfermagem secundárias centram–se no empoderamento parental, no apoio emocional, bem como na manutenção do vínculo familiar. Por sua vez, as intervenções terciárias incidem na ligação com os recursos comunitários.

## CONSIDERAÇÕES FINAIS

Com esta revisão escopo, foi possível realizar o mapeamento da evidência científica relativamente às intervenções de enfermagem promotoras da parentalidade durante a hospitalização da criança em situação de vulnerabilidade, identificando as situações de vulnerabilidade vivenciadas pela criança e família em situação de hospitalização, bem como as necessidades dos pais durante a hospitalização da criança em situação de vulnerabilidade.

Compreende–se que a hospitalização da criança em contexto de vulnerabilidade se constitua para a família como um momento de estresse, muitas vezes proveniente do fato de os pais não saberem como agir perante uma situação que nunca antes experienciaram, implicando, muitas vezes, uma crise de identidade parental com indefinição dos papéis dos pais relativamente ao que podem ou não fazer e/ou ao que os profissionais esperam que eles façam. Esta é uma situação difícil e complexa, pois os enfermeiros têm a responsabilidade de aumentar a autoconfiança dos pais para que eles consigam gerir as suas emoções e organizar–se perante esse cenário da hospitalização do filho. 

As situações de vulnerabilidade descritas estão de acordo, na sua maioria, com a hospitalização da criança. A prematuridade e o contato com opioides no período perinatal são identificados, ainda, como situações que acrescem vulnerabilidade.

Identificaram–se como principais necessidades dos pais a necessidade de suporte emocional, a validação das emoções experienciadas, a necessidade de um sistema de suporte e de transporte, e a necessidade de educação para a saúde. 

As intervenções de enfermagem promotoras da parentalidade durante a hospitalização da criança em situação de vulnerabilidade relacionam–se com a educação para a saúde, com o encorajamento e empoderamento dos pais na participação dos cuidados ao seu filho durante a hospitalização, e com o estabelecimento de relações terapêuticas.

Conclui–se que as intervenções de enfermagem identificadas de nível primário (com foco na prevenção da doença e na educação parental), de nível secundário (com foco na capacitação e no empoderamento parental, no apoio emocional e suporte parental durante a hospitalização, e na manutenção do vínculo familiar) e de nível terciário (com foco na ligação com os recursos comunitários fomentando o restabelecimento do sistema para o período pós–alta), assentes em um modelo de parceria de cuidados e em uma prestação de cuidados centrada na família, aparentam responder às necessidades dos pais. Torna–se, assim, possível promover a parentalidade incentivando o exercício da autonomia e empoderando os pais em situações de vulnerabilidade, como hospitalização de um filho, prematuridade e contato com opioides no período perinatal.

Considera–se que seja importante a realização de mais estudos que descrevam as intervenções de enfermagem promotoras da parentalidade em situação de vulnerabilidade acrescida se atentando à cultura e às diferentes configurações familiares, bem como tenham enfoque nos diferentes estádios de desenvolvimento da criança em todo o percurso da hospitalização infantil.

Com base nos resultados mapeados nesta revisão, recomenda–se que os enfermeiros adotem uma abordagem holística e centrada na família durante a hospitalização da criança em situação de vulnerabilidade. As intervenções devem abranger desde a promoção da educação parental até a capacitação para o exercício da parentalidade, através do suporte emocional, promoção do vínculo familiar e comunicação terapêutica. Além disso, é essencial que os enfermeiros incentivem a participação ativa dos pais nos cuidados ao filho, inclusive através do uso de tecnologias, para manter o contato em sua ausência, assegurando que as famílias estejam conectadas a recursos comunitários que possam oferecer apoio contínuo após a alta hospitalar. A implementação dessas práticas pode contribuir significativamente para o fortalecimento da parentalidade e para a recuperação e bem–estar da criança. A formação contínua dos enfermeiros nesse âmbito será relevante, contribuindo, certamente, para a melhoria da qualidade dos cuidados.

## Data Availability

Os dados da pesquisa estão disponíveis no corpo do artigo.
